# Valbenazine induced acute parkinsonism in a patient with alcohol use disorder and central pontine myelinolysis

**DOI:** 10.1002/pcn5.70282

**Published:** 2026-01-10

**Authors:** Yasuhito Nagai, Miyata Mamiko, Koichi Miyakawa

**Affiliations:** ^1^ Department of Psychiatry Juntendo University Graduate School of Medicine Tokyo Japan; ^2^ Department of Psychiatry Juntendo University Urayasu Hospital Urayasu Japan

**Keywords:** central pontine myelinolysis, parkinsonism, valbenazine, VMAT‐2 inhibitor, Wernicke encephalopathy

## Abstract

**Background:**

Valbenazine is used to treat dyskinesia and is generally associated with limited side effects; however, a few cases with the adverse effect of parkinsonism are reported in the literature. In this study, we report a case of valbenazine‐induced acute parkinsonism.

**Case Presentation:**

The patient was a 60‐year‐old male who suffered from depression and alcohol use disorder. When he was 50 years old, he was diagnosed with Wernicke's encephalopathy, central pontine myelinolysis‐induced parkinsonism, and dyskinesia. After remission, dyskinesia persisted and was diagnosed as tardive dyskinesia. The patient was treated with tetrabenazine and valbenazine at different times, and only valbenazine treatment induced acute parkinsonism.

**Conclusion:**

Valbenazine can cause acute parkinsonism, even in patients who have previously tolerated tetrabenazine, and especially in those with underlying basal ganglia lesions.

## INTRODUCTION

Strong evidence supports the efficacy of vesicular monoamine transporter 2 (VMAT‐2) inhibitors for the treatment of tardive dyskinesia. VMAT‐2 inhibitors interfere with monoamine uptake and storage, resulting in the depletion of monoamines (e.g., dopamine, noradrenaline, serotonin, and histamine), and thereby reducing dyskinesia.^1^ Tetrabenazine use is currently discouraged due to its side effects, which include depression and parkinsonism. As alternative treatments, valbenazine and deutetrabenazine have been developed, which offer the advantage of an increased half‐life.^1^ Valbenazine generally shows limited side effects—primarily somnolence, akathisia, and dry mouth—although a few cases presenting with parkinsonism as an adverse effect have been described in the literature.^1^, ^2^, ^3^, ^4^

Here we report our experience with a patient diagnosed with alcohol use disorder and central pontine myelinolysis (CPM), who developed acute parkinsonism due to treatment with valbenazine. This patient provided written informed consent for the publication of this case.

## CASE PRESENTATION

The patient in this case was a 60‐year‐old man. At 50 years old, he experienced a depressive mood and suffered from insomnia due to job‐related stress. As his boss often blamed him for worsening business performance, his alcohol consumption increased. When he arrived at our hospital, he was diagnosed with major depressive disorder and was prescribed mirtazapine. After 3 months of medication, his mood improved, and he stopped attending outpatient visits and changed jobs. However, 2 months later, his depressive mood returned, he began drinking alcohol all day, and sometimes he could not attend work. Three months after he changed jobs, he was spending most of his time drinking, and gradually began to develop marche à petit pas and dysphagia.

**Figure 1:**
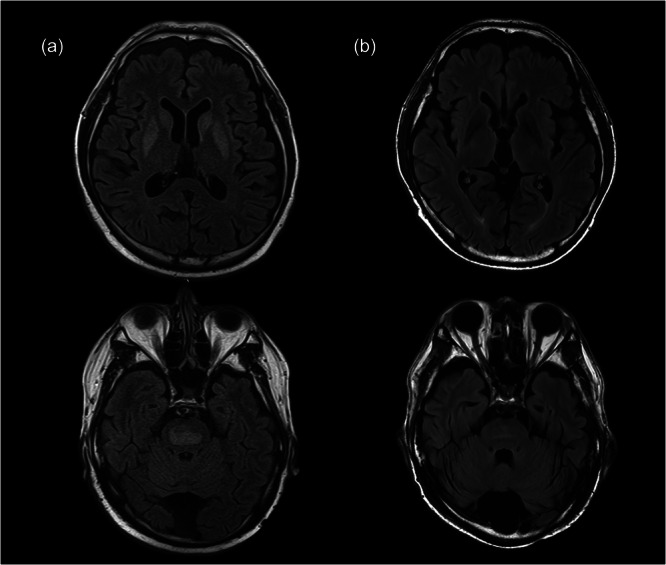
FLAIR images of this patient. (a) was performed in 2015 and (b) was performed in 2024. Hyperintensity in the basal ganglia and the central pons showed improvement.

At this point, he attended our hospital again (Day X). Laboratory serum testing revealed hyponatremia (117 mM/L) and hypokalemia (3.2 mM/L). Two weeks after his visit, he could not eat anything, and presented with somnolence, cogwheel rigidity, bradykinesia, and ataxia. He was admitted to our hospital on Day X + 2w. The hyponatremia was improved; however, laboratory testing revealed hypokalemia (3.1 mM/L) and a lower serum level of vitamin B1 (23 ng/mL), despite receiving 3 days of vitamin supplement therapy. Magnetic resonance imaging (MRI) showed symmetrical high intensity on the caudate nucleus, putamen, and pons (Figure 1). We diagnosed the patient with alcohol use disorder, Wernicke encephalitis, and CPM, and initiated treatment with thiamine injection 1500 mg/day and amantadine 200 mg/day. After 2 months of hospitalization, the patient still exhibited a weak voice, trunk ataxia, and dyskinesia. He was transferred to the rehabilitation hospital on Day X + 3m. After 1 month of rehabilitation (Day X + 4m), the patient still showed dyskinesia and mild dysarthria. The persistent dyskinesia caused anxiety, and he sometimes took lorazepam 0.5 mg and drank alcohol, although he started medication with acamprosate 1998 mg/day.

On Day X + 7m, he was referred to a neurologist. Laboratory testing and electroencephalography were normal, and the neurologist considered the possibility of a functional overlay. On Day X + 1y4m, after the patient stopped taking amantadine, the oral dyskinesia was worsened, and postural tremor was observed. He was treated with trihexyphenidyl 6 mg/day for 2 months, but this was not effective for his symptoms. On Day X + 2y1m, off‐label use of risperidone 0.5 mg was started, and the patient's dyskinesia was worsened. He was referred to another neurologist on Day X + 2y4m. He was diagnosed with tardive dyskinesia due to risperidone, and was prescribed tetrabenazine 12.5 mg/day for 2 months; however, this medication was not effective and did not cause parkinsonism.

Suffering from prolonged dyskinesia made the patient depressed and nervous. He continued to make outpatient visits to our psychiatry department. However, prescribed escitalopram, bromazepam, risperidone, quetiapine, and chlorpromazine were not effective for his depressive mood and anxiety. In particular, his dyskinesia transiently worsened while taking chlorpromazine. The treatment durations were 7 months on risperidone, 1 year and 5 months on quetiapine, and 3 years and 8 months on chlorpromazine. Ten years after the onset of CPM, he visited the Department of Neurology in our hospital. Neurological examination revealed tardive dyskinesia in the oral region, trunk, and limbs, without rigidity, and abnormal neural reflexes. MRI showed improvement in the basal ganglia and reduction of hyperintensity in the central pons (Figure 1).

We hypothesized that prolonged antipsychotic treatment might have caused tardive dyskinesia; we stopped all antipsychotics, but dyskinesia persisted. Next, we prescribed valbenazine 40 mg/day. After 2 days of medication, the dyskinesia in his limbs improved, but he could not stand alone due to postural instability. The next day, he could not eat because of worsening swallowing. After 7 days of taking valbenazine, he stopped this medication due to the side effect of parkinsonism. Three days after stopping valbenazine, the acute parkinsonism was ameliorated (on Day X + 10y3m).

## DISCUSSION

In this case, we could not clearly define whether the patient had Wernicke encephalopathy with CPM, or CPM with extra pontine myelinolysis. We could not confirm oculomotor dysfunction at the onset due to the patient's impaired consciousness at that time. However, the other symptoms supported the diagnosis of Wernicke encephalopathy. Wernicke encephalopathy and CPM are often complicated,^5^ and commonly co‐occur with many of the same conditions—including malnutrition, thiamine deficiency, hyponatremia, hypokalemia, and alcohol use disorder, which were observed in this case.^6^ The onset of parkinsonism may have occurred due to incomplete necrosis or cellular edema in the basal ganglia. Follow‐up MRI indicated that these changes were ameliorated and reversible, and liver function after discharge was within normal limits (Table 1). Thus, we diagnosed the prolonged dyskinesia symptoms as tardive dyskinesia induced by inappropriate long‐term antipsychotic medication. Valbenazine is generally considered to have superior pharmacokinetic properties and safety compared to tetrabenazine; however, in this case, valbenazine induced acute parkinsonism, while tetrabenazine did not. Valbenazine and tetrabenazine share an active metabolite, α‐dihydrotetrabenazine (α‐HTBZ), and their second active metabolites are NBI‐136110 and β‐HTBZ, respectively. All active metabolites inhibit VMAT‐2, but NBI‐136110 shows a lower affinity than α‐HTBZ.^7^ From the perspective of pharmacokinetics, valbenazine is generally better tolerated than tetrabenazine. Valbenazine interacts with CYP2D6 inhibitors and CYP3A4 inhibitors and inducers.^1^ Notably, in this case, brotizolam was the only prescribed medication related to CYP3A4 metabolism, and the side effect was not caused by a pharmacokinetic interaction. We only used the initial dose of both drugs. At the point of the ratio to the maximum dose, tetrabenazine seems to be a relatively low dose compared to valbenazine. We speculate that the patient's age may have contributed to the poor tolerance of valbenazine. The current literature includes four cases of valbenazine‐induced parkinsonism, in patients with ages of 73, 59, 44, and 69 years.^2^, ^3^, ^4^ These case reports may suggest that valbenazine‐induced parkinsonism should be considered as a potential adverse effect, particularly in middle‐aged or older patients.

**Table 1:** Liver function in blood test.

	Second visit	Hospitalization	Discharge	Referred to the neurologist		Administration of valbenazine
	X	X + 2w	X + 3m	X + 4 m	X + 6 y	X + 10y3m
AST (IU/L)	186	77	34	33	29	31
ALT (IU/L)	51	89	47	11	9	7
LDH (IU/L)	401	257	158	227	176	195
γGTP (IU/L)	369	159	52	38	28	21
PLT (10^4^/μL)	13.5	40.7	37.9	32.1	27.6	31.8

Abbreviations: ALT, alanine aminotransferase; AST, aspartate aminotransferase; LDH, lactate dehydrogenase; plt, platelet; γ‐GTP, γ‐glutamyl transferase.

## CONCLUSION

Valbenazine can cause acute parkinsonism, even in patients who previously tolerated tetrabenazine, and especially in those with underlying basal ganglia lesions.

## AUTHOR CONTRIBUTIONS

All authors treated the patient. Yasuhito Nagai wrote the original manuscript. All authors participated in the discussion, writing this manuscript, and revisions. All read and approved the final version of the manuscript.

## CONFLICT OF INTEREST STATEMENT

The authors declare no conflicts of interest.

## ETHICS APPROVAL STATEMENT

N/A.

## PATIENT CONSENT STATEMENT

The patient provided written informed consent for the publication of this case.

## CLINICAL TRIAL REGISTRATION

N/A.

## Data Availability

Data sharing is not applicable to this article as no datasets were generated or analyzed during the current study.
